# Quantitative analysis of the reversibility of knee flexion contractures with time: an experimental study using the rat model

**DOI:** 10.1186/1471-2474-15-338

**Published:** 2014-10-07

**Authors:** Guy Trudel, Hans K Uhthoff, Louis Goudreau, Odette Laneuville

**Affiliations:** Department of Medicine, Faculty of Medicine, University of Ottawa, 451 Smyth Rd, Ottawa, ON K1H 8M5 Canada; Bone and Joint Research Laboratory, Faculty of Medicine, University of Ottawa, 451 Smyth Rd, Ottawa, ON K1H 8M5 Canada; Division of Orthopedic Surgery, Faculty of Medicine, University of Ottawa, 451 Smyth Rd, Ottawa, ON K1H 8M5 Canada; Biomedical Engineering, The Ottawa Hospital Rehabilitation Centre, 505 Smyth Road, Ottawa, ON K1H 8M2 Canada; Department of Biology, Faculty of Science, University of Ottawa, 30 Marie Curie, Ottawa, ON K1N 6N5 Canada

**Keywords:** Contracture, Knee joint, Biomechanics, Reversibility, Temporal study

## Abstract

**Background:**

Knee flexion contractures prevent the full extension of the knee joint and cause disability. The etiology is not well defined. Extended periods of immobilization of joints lead to contractures difficult to completely reverse by rehabilitation treatments. Recovery of the complete range of motion without intervention has not been studied but is of importance to optimize clinical management. This study was designed to quantify the spontaneous reversibility of knee flexion contractures over time.

**Methods:**

Knee flexion contractures of increasing severities were induced by internally fixing one knee of 250 adult male rats for 6 increasing durations. The contractures were followed for four different durations of spontaneous recovery up to 48 weeks (24 groups, target n = 10 per group). The angle of knee of extension at a standardized torque was measured. Contralateral knees constituted controls.

**Results:**

Full reversibility characterized by knee extension similar to controls was only measured in the lowest severity group where 4 weeks of spontaneous recovery reversed early-onset contractures. Spontaneous recovery of 2, 4 and 8 weeks caused partial gain of knee extension in longer-lasting contractures (P ≤ 0.05; all 4 comparisons). Extending the durations of spontaneous recovery failed to further improve knee extension (P > 0.05, all 12 comparisons). No reversal occurred in the highest severity group (32 week; P > 0.05).

**Conclusions:**

Reversibility of knee flexion contractures was dependent on their severity. Full spontaneous recovery was limited to the least severe contractures. While contractures initially improved, a plateau was reached beyond which additional durations of spontaneous recovery led to no additional gain of knee extension. These results support our view that without treatment, permanent losses in knee mobility must be anticipated in immobility-induced contractures.

**Electronic supplementary material:**

The online version of this article (doi:10.1186/1471-2474-15-338) contains supplementary material, which is available to authorized users.

## Background

A contracture limits the passive range of motion of a joint and is caused by multiple factors that include joint immobility [1]. Contractures are prevalent clinically as a consequence of casting, joint arthroplasty, sports injuries, bed rest and others [2–6]. Once established, joint contractures can limit function and performance [1].

Just how effective is spontaneous recovery in returning a contractured joint to normal range of motion? The recovery potential of joint contracture has been the focus of little research, restricted to animal experimentation of which very few studies reported quantitative data measured over time [7]. In addition, published reports are controversial; some investigations in a rabbit model proposed that contractures left untreated may be fully reversible [8, 9]; this view was recently echoed [10]. Other studies suggest the contrary: only contractures of recent onset have the potential to recover without treatment in the rat, rabbit and horse [11–15]. The potential for reversibility of joint contractures needs to be established.

Determining the reversibility of joint contractures in an animal model can have clinical relevance for patient care and for resource utilization. If contractures are largely reversible, treatment is not justified. If largely irreversible, delays in diagnosis or treatment may be costly since currently, there is no effective medical treatment to reverse or cure long-lasting joint contractures. For these reasons, a comprehensive reversibility experiment using a rat model and including quantitative measures of ROM over a time course would provide the rationale to improve the management of joint contractures.

In the current study, knee joint flexion contractures of various severities underwent incremental durations of spontaneous recovery in a rat model. The range of knee extension was measured using an automated goniometer. Our objectives were 1) to determine whether knee joint contractures are fully reversible during recovery without treatment and 2) to determine the duration of spontaneous recovery that produces maximal partial reversibility of joint contractures. Our hypotheses were that 1) while early-onset knee flexion contractures may be reversible, long-lasting contractures do not fully reverse spontaneously and 2) while initial partial reversibility may occur, extending the duration of recovery does not lead to further gains in knee extension.

This study provides evidence-based guidelines on the timing of intervention to manage joint contractures of various severities by accurately predicting their natural course.

## Methods

**Figure 1:**
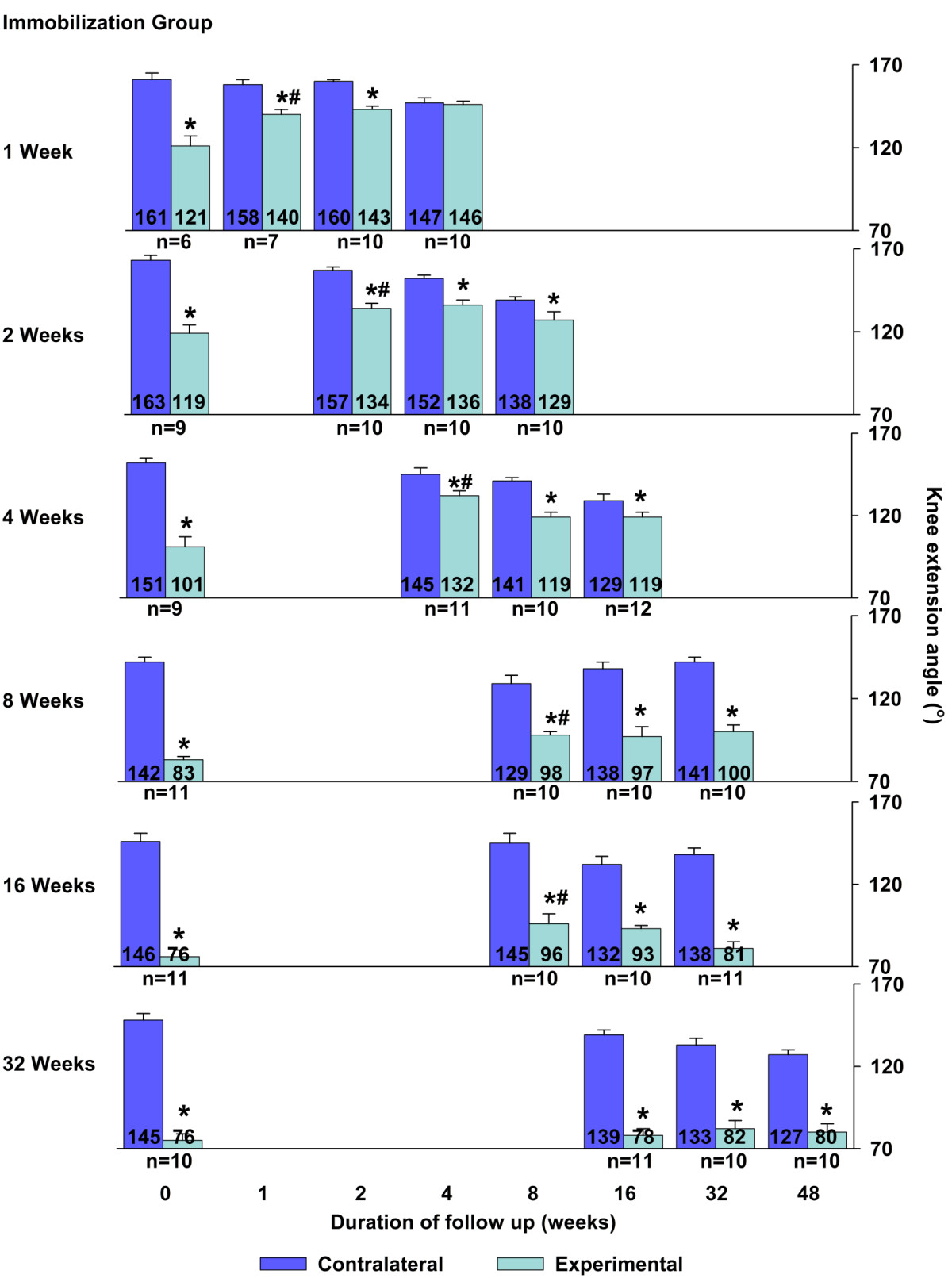
**Study design with internal fixation and spontaneous recovery durations and sample size per group.** Angle of extension in rat knee joints for the 24 groups at torque = 12.5 N-cm. *P ≤ 0.05 for difference between the mean ranges of knee extension of contracture versus contralateral knees. All P > .001 identified. #*P* ≤ 0.05 for gain in knee extension angle after recovery duration half, equal to, double, or quadruple the duration of surgical fixation compared to the previous duration. Error bars = 1 standard error of the mean.

This project was approved by the University of Ottawa Animal Care Committee. Rat knee contractures of various severities were produced by extra-articular fixation of one knee of 250 adult male Sprague–Dawley rats, each weighing 325 g, aiming for a final sample size of 40 each for six durations: 1, 2, 4, 8, 16 or 32 weeks [7]. Briefly, under general anesthesia and alternating right and left legs, a Delrin® plate was surgically fixed with screws to the proximal femur and distal tibia. This surgical internal fixation spanned the knee joint, away from all intra-articular knee structures while achieving rigid fixation in 45° of flexion [7]. Preoperatively the rats received slow-release buprenorphine and ketamine. Bupivacaine hydrochloride was applied transdermally at closure. Rats had access to food ad libitum and their activity was unrestricted in cages. Leg pain beyond 4 days post-op was treated with gabapentin s/c for 7 days. Nonsteroidal anti-inflammatory medications were avoided. Wound infections were treated with amoxicillin trihydrate/clavulanate potassium orally twice daily for 7 days; fluoroquinolones were avoided. More animals were operated in experimental groups of greater severity in anticipation of attrition. Animals with surgical failures or requiring euthanasia ahead of endpoint were replaced.At the end of the fixation period, the plate and screws were removed; also, any fibrous tissue covering the plate was divided at the proximal femur and distal tibia. The procedure created knee flexion contractures of various levels of severity depending on the duration of fixation. The rats were assigned to 1 of 4 durations of spontaneous recovery (approximately 10 per duration; Figure 1). One group was killed immediately after plate removal and represented the contracture with no recovery. All groups had one duration of recovery equal to that of fixation (Figure 1). For the first 5 durations of fixation (1, 2, 4, 8 and 16 weeks) one group had a duration of recovery double the duration of fixation (Figure 1). Finally, for the first 4 durations of fixation (1, 2, 4 and 8 weeks), one group had a duration of recovery four times that of the duration of fixation (Figure 1). We could not double the duration of recovery of knees surgically fixed for 32 weeks or quadruple the duration of recovery in animals fixed for 16 and 32 weeks owing to the 2-year life expectancy of the Sprague–Dawley rat. Therefore, for these 2 durations, spontaneous recovery period equal to half the duration of fixation was studied (Figure 1).

**Figure 2:**
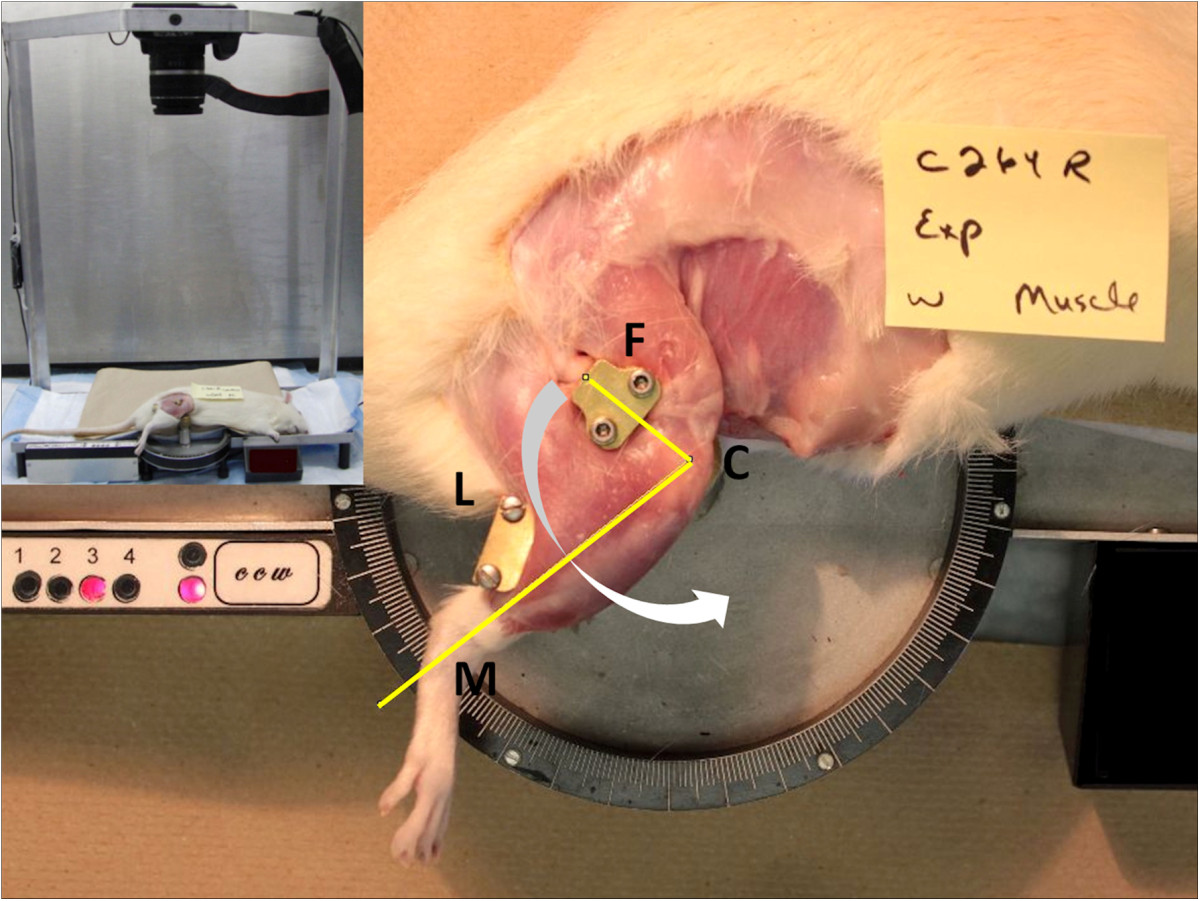
**Arthrometer used to measure angle of extension.** The right femur is fixed in a grooved metal clamp (F). The lateral condyle is positioned at the center of rotation (C). A motor-driven movable arm with two uprights posts pushes the posterior leg (L) into extension at a fixed distance from the center of rotation and at a predetermined speed. At torque = 12.5 N-cm a picture is taken of the knee (left upper corner insert shows the camera mounted above the arthrometer). The femorotibial angle is drawn using the femoral line from lateral condyle (C) to the middle of the femur clamp (F) (femoral diaphysis) and the tibial line from the lateral condyle (C) to lateral malleolus (M). The femorotibial angle corresponds to the angle of extension reached by the knee, with full extension defined at 180°. Once the rat leg is positioned, joint angle measurement is investigator-independent.

The knee contractures were not complicated by factors such as intra-articular trauma or change in neurological status. The joints received no physical intervention after fixation removal: the data correspond to spontaneous or untreated recovery of knee contracture.At endpoint, the rats were killed by administration of carbon dioxide. The knee angle of extension was measured using a motor-driven arthrometer (Figure 2). The animal was positioned on its side with the experimental leg facing upwards. The femur was secured in a grooved metal clamp. The lateral condyle was adjusted over the center of rotation of the arthrometer. A movable arm with two upright posts positioned behind the leg pushed it into extension with a force of 426 g applied at a distance of 30 mm of the center of rotation. The initial speed of rotation of 6.6 RPM was gradually slowed down until a torque of 12.5 N-cm was reached. This torque was selected because it achieves complete knee extension in normal rats. The motor then stopped while a camera (Canon EOS-500D, 30–2, Shimomaruko 3-chome, Ohta-ku, Tokyo 146–8501, Japan) attached to the frame of the arthrometer was triggered to take a picture (Figure 2). The rigid fixation of the arthrometer and the force applied horizontally optimized the acquisition of true lateral images without distortion. The animal was then turned onto its other side, and the same measures were repeated on the contralateral knee. The knee joint angle measurement was fully automated and operator-independent. In this study, full knee extension was defined as 180°.Knee images were analyzed by the same person, blinded to the experimental set-up of the animal. Images were opened with ImageJ version 1.45 s (National Institutes of Health, Bethesda, Maryland) and calibrated. The two arms of the femorotibial angle were drawn using the angle tool. The femoral line was drawn from the lateral condyle to the middle of the femur clamp (aligned with the femoral diaphysis) (Figure 2). The tibial line went from the lateral femoral condyle to the lateral malleolus (Figure 2). The femoro-tibial angle corresponded to the maximal angle of knee extension reached at the preset torque.

### Statistical analyses

We used SPSS 20.0 (IBM, Armonk, New York) for statistical testing. The data was compared in two ways. First, to detect a difference in mean knee range of extension between the experimental and contralateral knees; we ran 1-tail *t*-tests for the 24 experimental groups assuming the experimental knees would have a lower mean angle of extension than contralateral knees. A *P* value of ≤0.05 was interpreted as statistically significant: contractures had not fully reversed. Second, to detect the duration of recovery that improved contracture: the mean angle of extension of the experimental knees after each duration of recovery was compared to that at the previous duration of recovery using univariate analyses (recovery 1 vs 0, 2 vs 1, and 3 vs 2). A *P* value of ≤0.05 after Bonferroni correction (given the same rats were included in two different analyses) was interpreted as statistically significant: a plateau had not been achieved and this additional duration of recovery improved the contracture. Third, we tested for a change with time in the contralateral knee using an ANOVA for each contracture severity; a *P* value of ≤0.05 was interpreted as statistically significant. A confidence level of 95% was used on all analyses.

## Results

Thirteen rats required local wound care, of which 12 received antibiotics; all 13 were treated and included. At endpoint, data for 12 animals were not analyzed for persistent fibrous adhesions, leg fracture during testing, extension angle over 195° or images not recorded. Final number of experimental animals was 238; the distribution per group, fixation and recovery durations are shown in Figure 1. The animal model created contractures of various severities with maximal knee extension reaching 121° (101–140), 119° (96–133), 101° (77–136), 83° (75–93), 76° (60–103) and 76° (59–94) after 1, 2, 4, 8, 16 and 32 weeks of internal fixation, respectively (P = .003 for first comparison and <0.001 for the remaining 5 comparisons to contralateral knees; Figure 1).Full reversal occurred after 4 weeks of recovery in 1-week-old contracture. At all other durations of recovery, the mean angle of knee extension was smaller than contralateral knees (Figure 1). Thus, except for 1-week-old contractures, a full spontaneous reversal of the knee contracture was not observed in any joint contracture.

Durations of recovery that produced partial reversal of knee contracture were identified. Recovery for 2, 4, 8 and 8 weeks led to partial reversal of the contracture caused by 2, 4, 8 and 16 weeks of fixation, respectively (respective p-values of: .052, <.001, .048 and .009; Figure 1). The extent of partial gain was 15°, 31°, 15° and 20° for initial contractures of 44°, 40°, 59° and 70° respectively (Figure 1). These constituted a plateau after which additional durations of recovery added no significant gain in knee extension compared with the previous recovery duration (*P* > 0.05 for all 8 comparisons; Figure 1). Specifically, doubling the duration of recovery added no knee extension compared to the duration of recovery equal to the duration of fixation in all applicable groups (*P* > 0.05 for all 4 comparisons; Figure 1), and quadrupling the duration of recovery, when feasible, added no gain in knee extension compared to recovery double the duration of fixation (*P* > 0.05 for all 3 comparisons; Figure 1).

Recovery durations of 16, 32 or 48 weeks after 32 weeks of surgical fixation did not change the mean angles of knee extension (*P* > 0.05 for all 3 comparisons; Figure 1).Knees contralateral to the experimental knees showed a decrease in angle of extension over the duration of recovery for groups with unilateral fixation durations of 1, 2, 4 and 32 weeks (respective p-values of: .002, <.001, p < .001 and p = .005; Figure 1).

## Discussion

**Figure 3:**
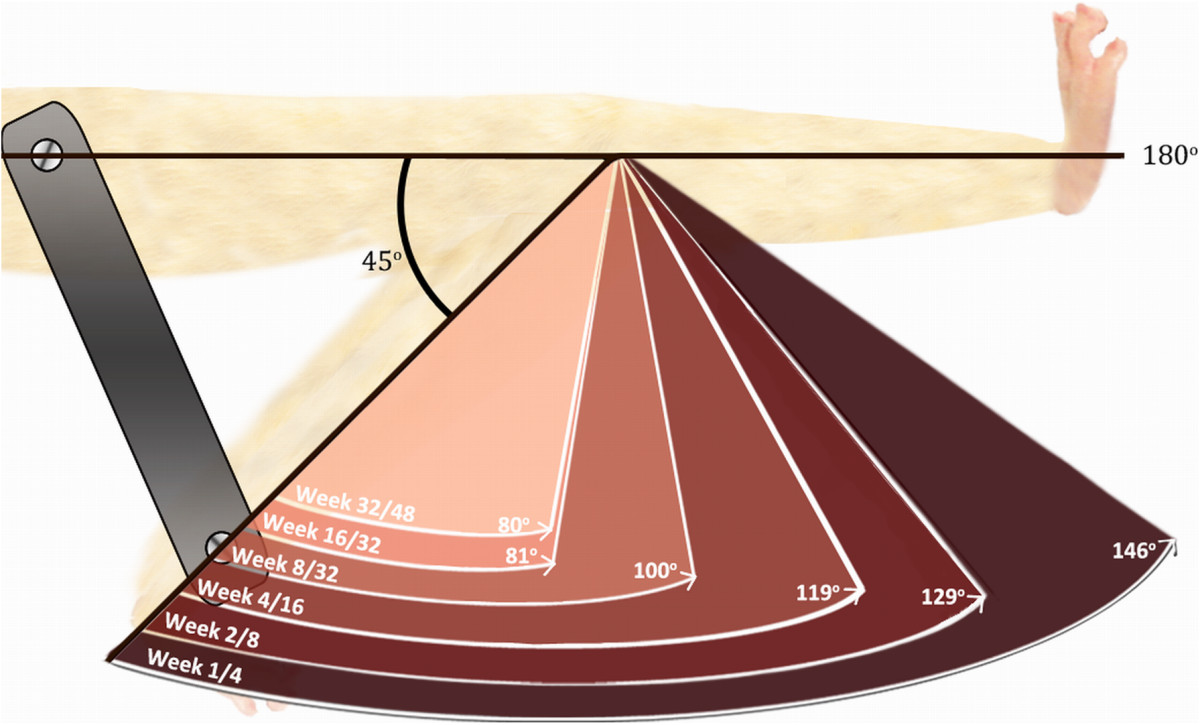
**Range of extension after spontaneous recovery of knee joint contractures of various severities.** Knees were surgically fixed at approximately 45° of flexion. More severe flexion contractures obtained decreased range of knee extension, irreversibly, despite proportionately long recovery durations. Data shown correspond to the angles of extension reached after the longest spontaneous recovery durations. (e.g., “week 2/8” = 2 weeks of fixation and 8 weeks of recovery).

Prolonged immobility of healthy joints resulted in contractures in the rat model. We quantified the spontaneous reversibility of knee joint contractures of various severities in 24 situations over 80 weeks. The data constitute convincing evidence for dose–response relationships not only between severity of the knee contracture and duration of internal fixation but also between severity of the contractures and potential for reversibility with spontaneous recovery (Figure 3).

Spontaneous recovery of rat knee contractures for 4 weeks allowed full reversal of 1-week-old contractures. At all other contracture severities, knee flexion contractures were not fully reversible no matter the duration of recovery. These results confirm the first hypothesis that only recent-onset knee flexion contractures fully reverse spontaneously. This study confirmed reports of incomplete reversal of joint contractures in various animal models [11–16] and improved upon previous investigations by the broad range of 18 clinically relevant durations of spontaneous recovery, from adult to geriatric age, by standardized mechanical testing, and by sufficient sample size for statistical testing. These data should help resolve the controversy regarding the potential for full spontaneous recovery of joint contractures secondary to immobility [8, 10].Within a specific time window — recovery for 2–8 weeks, some knee joint contractures were partially reversible. The extent of partial reversal was modest, an average gain of 20° of knee extension. This corresponded to an average 41% of the contractures (Figure 1). These constituted plateaus in the spontaneous recovery; extending the duration of recovery never led to a significant improvement of the contractures, which confirms the second hypothesis. Once a joint contracture is diagnosed, the current study implies that simple observation is not an appropriate option. Our data predict a poor prognosis, with recovery of only 20° (or 41%) of the contracture. Therefore intervention may be necessary to regain knee extension beyond natural recovery.

This study also measured decreased knee extension over time in the rat knees contralateral to a contractured knee (Figure 1). In this animal model, at least three factors can contribute: both surgical procedures involved general anesthesia and postoperative recovery (approximately 1 week each). The temporary general hypomobility may have contributed to the loss of extension in contralateral knees. Secondly, an index contracture in the experimental leg may limit extension in contralateral knees to smoothen the gait pattern; patients with osteoarthritis and a unilateral knee flexion contracture lacked knee extension of the contralateral knee [17]. Thirdly, aging remains a controversial contributor to decreased range of motion in diarthrodial joints [18–24]. In this study, using the contralateral knee joint for comparison controlled for the postoperative general hypomobility and for aging.

This animal model studied the simple immobility-induced joint contractures. No articular trauma, osteochondral damage or hemarthrosis accompanied the immobilization. No neurological injury altered the limb muscle tone (increased or decreased tone with an upper or a lower motor neuron injury, respectively) Importantly, no treatment was provided. This study produced normative data on the effects of joint immobility. The effects of other variables (partial mobility, articular trauma, change in neurological status, various treatments) need to be studied separately. Similarly, distinguishing the tissue limiting the knee joint, articular or muscular, can be obtained by comparing range of extension before and after myotomies [25].

The range of extension of the knee is a key variable in the biomechanical gait assessment. Full knee extension reaching 180 degrees is necessary for normal walking in humans. The magnitude of the knee flexion contracture proportionately disturbs the gait pattern. In humans, knee joint contractures required more quadriceps force for stability, caused a shorter stride length, increased oxygen consumption and negatively impacted balance and risk of falling [26–28]. For some patients, the immobility of normal joints is temporary (e.g., brace, cast, intensive care stay), and eventually they will actively use their joints again. The current study in the rat model shows that a statistically significant lack of knee extension remained despite long periods of spontaneous recovery similar to patients with fixed knee flexion contractures (Figure 3). The quantitative results measured over a time course of unassisted recovery in the current study may apply to patients with knee flexion contracture caused by prolonged immobility: after 2 weeks of immobility, their joints may develop a contracture that will not be fully reversible without treatment [3].

In the current study, 2 weeks of knee joint fixation had already caused contractures, not completely reversible without intervention. Early surveillance and intervention may decrease the risk of contractures [29]. A survey found that, of patients spending 2 weeks or more in an intensive care unit, 26% never had their joint motion documented [30]. Similar to the current experimental results, the development of contractures was linked to the duration of immobility: patients staying 8 weeks or longer in intensive care had an adjusted odds ratio of 7.1 to 1 of presenting a joint contracture compared to patients staying for 2–3 weeks [3]. The current study confirmed that the longer a joint contracture remains undiagnosed or untreated, the more severe and irreversible the structural changes [31].

The animal model differed from clinical practice in that a brace or a cast causes less rigid immobility than the extraarticular fixation. Clinically, spasticity or musculoskeletal lesions often accompanies joint contractures in stroke, spinal cord injury, peripheral nerve injuries, trauma [2, 6]. Finally, in clinical practice some form of treatment may be instituted when a contracture is diagnosed.

### Study limitations

First, the quadruped gait of the rat permitted long-term tolerance of the knee fixation in flexion because they never walk with their knee in full 180° of extension. However, functionally, a knee extension deficit in the rat is different than in humans [27]. Whereas the basic mechanical data from this study may apply to other diarthrodial joints, the functional impact of contractures varies from joint to joint. Second, we did not study the effect of recovery beyond a fourfold duration of fixation, because we had already reached the life expectancy of some animals. Whether extended periods of recovery would have been beneficial is unlikely, since doubling or quadrupling the duration of recovery did not lead to any significant gain in knee extension. Finally, short durations of recovery after long periods of fixation may have shown that the plateau in partial reversal was achieved earlier than after 8 weeks of recovery.

## Conclusions

In this animal model knee flexion contractures of 2-week onset or longer did not fully recover when untreated. Spontaneous recovery initially allowed modest, partial range of knee extension before a plateau was reached; extending the duration of spontaneous recovery was an ineffective intervention. In the absence of treatment, permanent losses in knee extension must be anticipated.

## Authors’ original submitted files for images

Below are the links to the authors’ original submitted files for images. Authors’ original file for figure 1 Authors’ original file for figure 2 Authors’ original file for figure 3
