# Supplementary figures and images for: Quantitative analysis of the reversibility of knee flexion contractures with time: an experimental study using the rat model
Source: BMC Musculoskelet Disord. 2014 Oct 7;15:338. doi: 10.1186/1471-2474-15-338 (PMC4289348; doi:10.1186/1471-2474-15-338)

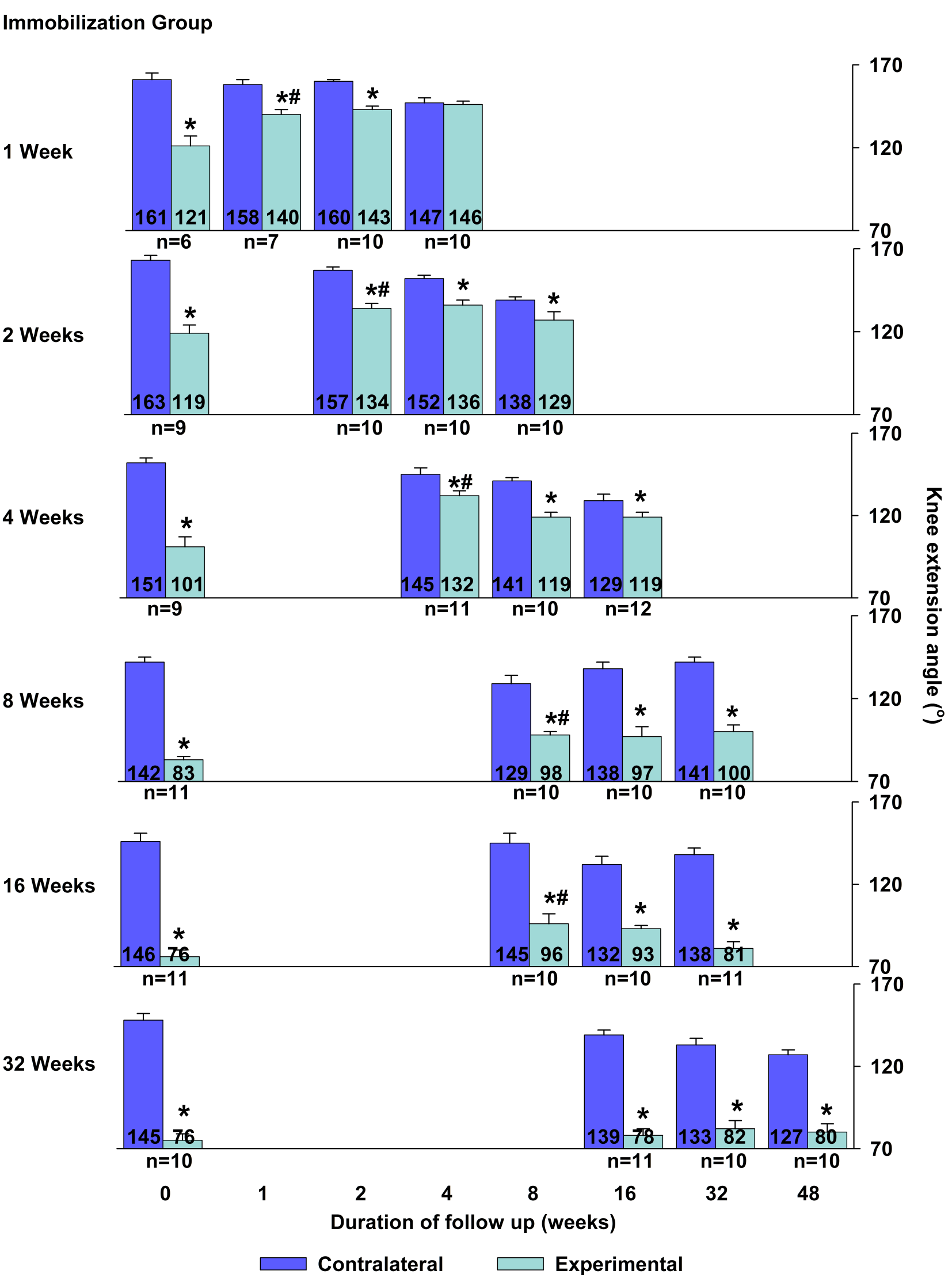

Supplement: Supplementary file 1 — Authors’ original file for figure 1 [file 12891_2014_2373_MOESM1_ESM.tif]

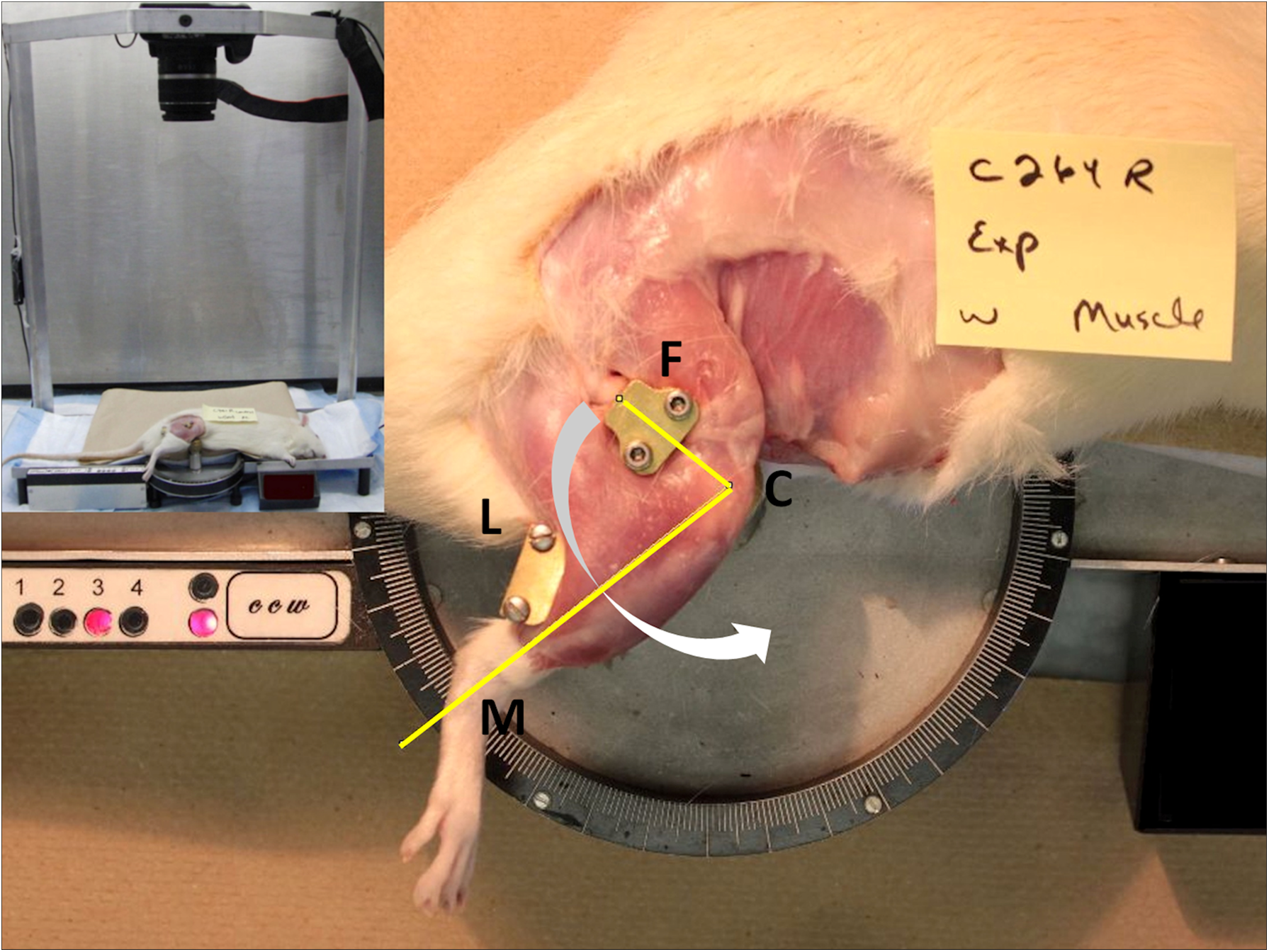

Supplement: Supplementary file 2 — Authors’ original file for figure 2 [file 12891_2014_2373_MOESM2_ESM.tiff]

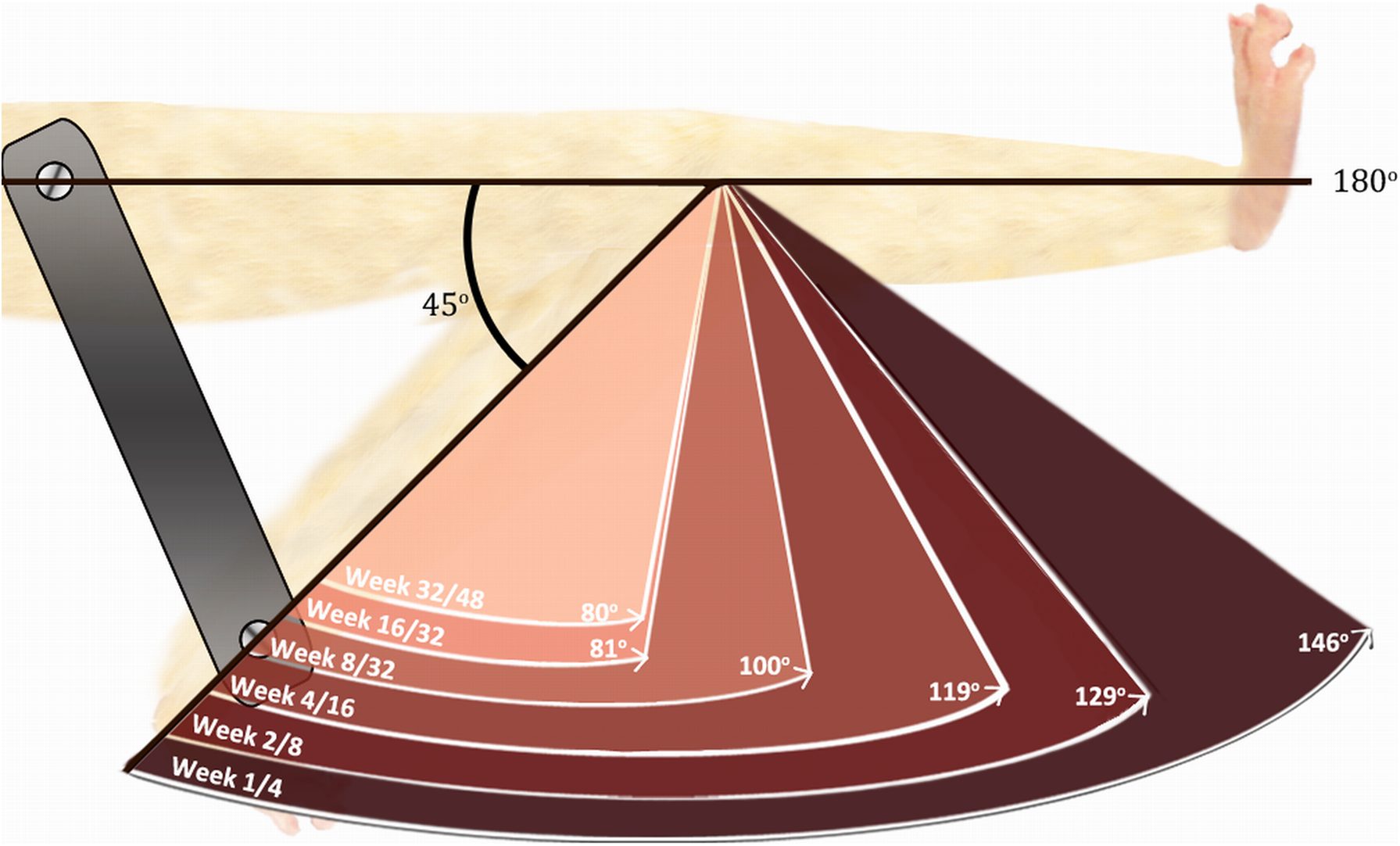

Supplement: Supplementary file 3 — Authors’ original file for figure 3 [file 12891_2014_2373_MOESM3_ESM.tiff]
